# Analysis and prediction of the burden of lung cancer attributable to diet low in fruits in China and the global from 1990 to 2021

**DOI:** 10.1371/journal.pone.0342584

**Published:** 2026-03-11

**Authors:** Caifa Ji, Youjian Yao, Mei Gui

**Affiliations:** School of Public Health, Hainan Medical University, Haikou, Hainan, China; Debre Tabor University, ETHIOPIA

## Abstract

**Objectives:**

To analyze the trend of the disease burden of lung cancer attributable to diet low in fruits among the Chinese and the global populations from 1990 to 2021, describe the disease burden situation in 2021, and predict the development trend of the disease burden attributable to diet low in fruits over the next 25 years, so as to provide scientific suggestions for the prevention and control of lung cancer.

**Methods:**

The paper utilized data from the Global Burden of Disease Study 2021 (GBD 2021). The joinpoint regression model was employed to calculate the annual percentage change (APC) and the average annual percentage change (AAPC) to assess the changing trend of the burden of lung cancer attributable to diet low in fruits. The disease burden of lung cancer attributable to diet low in fruits was predicted for the next 25 years using a Bayesian age-period-cohort (BAPC) model.

**Results:**

From 1990 to 2021, the number of mortality and disability-adjusted life years (DALY) of lung cancer attributable to diet low in fruits in China and the global increased significantly, while the age-standardized rates decreased significantly. In China, the estimated annual percentage change (EAPC) in the total population and different gender categories ranged from −4.0 to −2.8. The mortality number of lung cancer attributable to diet low in fruits in China increased with age, reaching a peak at 70−74 years. Similarly, the age-standardized DALY rate paralleled mortality rate trends across genders and age groups. The AAPC in age-standardized mortality and DALY rates were −2.69 and −3.15, respectively. According to the BAPC model prediction results that by 2046, the age-standardized mortality and DALY rates of lung cancer attributable to diet low in fruits in China and the global will decrease by 31.58%, 24.68%, 29.28%, and 24.34%, respectively.

**Conclusions:**

From 1990 to 2021, the mortality and DALY rates of lung cancer attributable to diet low in fruits in China and the global both decreased. The disease burden of lung cancer attributable to diet low in fruits in male has always been higher than that in female, and the mortality and DALY rates were the highest among the elderly. It is expected that by 2046, the mortality and DALY rates of lung cancer attributable to diet low in fruits will further decrease.

## 1. Introduction

Lung cancer is among the most prevalent cancers globally and remains a leading cause of cancer-related mortality [1–4]. According to the most recent data released by the National Cancer Center of the United States in 2022, China reported 1,060,600 new cases and 733,300 mortality from lung cancer, both of which rank first among all cancer types [5]. In the past few years, the incidence and mortality rates of lung cancer in China have been increasing, with notable disparities observed across different genders, age groups, and regions [6]. The incidence and mortality of lung cancer are determined by multiple risk factors. In addition to smoking, air pollution, and genetic predisposition, dietary factors also play a crucial role in the development and progression of lung cancer [7,8]. Multiple meta-analyses have demonstrated that dietary risk factors contribute to an increased likelihood of developing lung cancer [9–12]. A large prospective cohort study has demonstrated that a moderate intake of fruits, vegetables, breakfast cereals, and dietary fiber is associated with a reduced risk of lung cancer. The findings suggest that consuming 100 grams of fruit per day can decrease the risk of lung cancer by 10%, whereas excessive consumption of red and processed meat may elevate the risk [13]. According to the “Scientific Research Report on the Dietary Guidelines for Chinese Residents (2021)”, the problem of chronic diseases related to diet is becoming increasingly serious. Consuming 200 ~ 350g of fresh fruits daily has been associated with a 24% reduction in the risk of lung cancer [14,15]. Other factors, including air pollution, diet low in fruit, elevated fasting blood glucose levels, and exposure to occupational carcinogens, not only independently contribute to an increased risk of lung cancer but may also interact with smoking, exerting a significant influence on the epidemiology of the disease [1].

Assessing the burden of lung cancer attributable to diet low in fruits is essential for policymakers in developing effective national cancer control strategies. Implementing dietary modifications in conjunction with a healthy lifestyle may further mitigate the risk of lung cancer [16]. Fruits and vegetables that are rich in beta-carotene, carotenoids, vitamin C, and isoflavones have been shown to reduce the risk of lung cancer [17]. In contrast, the consumption of red meat, processed meat, alcohol, and both excessive and diet low in fruits may contribute to an increased risk of lung cancer [17,18]. Previous research has extensively examined the burden of lung cancer in China attributable to smoking [19,20], and air pollution [4,21–23]. However, relatively few studies have focused on the disease burden of lung cancer associated with dietary risks, particularly diet low in fruits [13,24–26]. Therefore, this study aims to analyze the current status, temporal trends, and future projections of the disease burden of lung cancer attributable to diet low in fruits. Additionally, it seeks to provide recommendations for lung cancer prevention and control. These findings are crucial for optimizing prevention strategies and improving the allocation of healthcare resources.

## 2. Data and methods

### 2.1. Data source and study design

The data is sourced from the GBD 2021 database, which is a free public database jointly initiated by the World Bank (WB), the World Health Organization (WHO), and other institutions. All data are open access and freely available for public use. It contains data on 371 diseases and injuries and 88 major risk factors in 204 countries and regions. This study extracted the mortality number (cases), mortality rate (per 100,000), DALY number (person-years), and DALY rate (per 100,000) of lung cancer attributable to diet low in fruits in China and the global, from 1990 to 2021, stratified by gender and age groups, for analysis. The age groups in this study were divided every five years, with those over 95 years as a separate group, including “25-29 years”, “30-34 years”, “35-39 years”, “40-44 years”, “45-49 years”, “50-54 years”, “55-59 years”, “60-64 years”, “65-69 years”, “70-74 years”, “75-79 years”, “80-84 years”, “85-89 years”, “90-94 years”, and “95+ years”, totaling 15 age groups.

The population attributable fraction (PAF) is a fundamental metric for evaluating disease risk factors and quantifying the proportion of disease burden attributable to specific exposures. Within the GBD framework, exposure levels are typically estimated using modeling approaches such as Spatiotemporal Gaussian Process Regression (ST-GPR) and DisMod-MR, which enable an accurate characterization of the associations between risk factors and health outcomes. The resulting PAF estimates are subsequently applied to derive attributable DALY, year of life lost (YLL), and year lived with disability (YLD). In the GBD 2021 cycle, “diet low in fruits” was designated as a major component of dietary risk factors, and its corresponding PAF was utilized to evaluate its contribution to the burden of lung cancer [27]. In the GBD framework, estimates of disease burden attributable to specific risk factors are derived from model-based associations. Accordingly, in this study, the phrase “lung cancer attributable to diet low in fruits” reflects an association inferred from an ecological modeling approach, rather than a direct causal estimate.

### 2.2. Ethical approval and consent to participate

This study adheres to the ethical principles of the Declaration of Helsinki and complies with relevant local laws and regulations. As all the data used in the study are from publicly accessible databases, no review by an institutional ethics committee is required.

### 2.3. Statistical analysis

This study employs the EAPC to quantify the trends of age-standardized mortality rate (ASMR) and age-standardized DALY rate (ASDR) of lung cancer. The calculation formula is: The EAPC value is $(e^{\beta }-1ast100\%$, where is obtained through linear regression. When the estimated EAPC value and its 95% confidence interval (95%CI) are both greater than 0, the ASMR or ASDR is considered to be on an upward trend. Conversely, if the estimated EAPC value and its 95%CI are both less than 0, ASMR or ASDR is regarded as a downward trend. If both the upper and lower limits are 0, it indicates a stable trend. The joinpoint regression model (Joinpoint version 5.1.0.0 was used to create this model) is a statistical method used to analyze the trend changes in time series data. Its core functions include calculating the APC and AAPC. To further reflect the trend changes in the disease burden of lung cancer, this paper uses the joinpoint regression model to calculate the APC and AAPC. It analyzes the changing trend of lung cancer mortality and DALY rates attributable to diet low in fruits over time. Log-transformed age-standardized indicators were fitted using a regression model of the form ln(y)= α+ βx+ ε, where y denotes the age-standardized measure of interest and x represents the calendar year. The AAPC was then derived as 100*(exp(β)−1), with corresponding 95%CI computed directly from the model [28]. In this study, the grid search method (GSM) was applied to enumerate all feasible joinpoint configurations for the segmented regression model, and the sum of squared errors (SSE) and mean squared errors (MSE) were computed for each candidate specification. The configuration yielding the smallest MSE was selected as the final set of joinpoints, after which the corresponding model parameters ($\beta_{\text{0}}$, $\beta_{\text{1}}$, $\delta_{\text{1}}$, …, $\delta_{k}$, etc.) were estimated. The optimal number of joinpoints was subsequently determined using Monte Carlo permutation testing, with the permissible range constrained to 0 ~ 5 to ensure adequate model fit while mitigating potential overfitting. The principal statistical outputs included APC, AAPC, and their respective 95%CI. Among them, APC describes the speed of change in the disease burden of lung cancer within a specific time period, and AAPC describes the overall trend change amplitude of the disease burden of lung cancer. If AAPC is positive, it indicates an overall upward trend; if it is negative, it indicates a downward trend; if it is close to 0, it indicates that the trend change is not significant. If APC = AAPC, it suggests that no turning point was found throughout the entire time span; if the 95%CI does not include 0, the trend change is statistically significant. Decomposition analysis is a methodological approach employed to elucidate patterns and temporal changes within data. It disaggregates variations in disease incidence over a given period into contributing components such as changes in population age structure, population growth, and epidemiological trends, thereby facilitating a clearer understanding of the relative influence of each factor on the overall observed changes. The decomposition analysis formula, using DALY as an example, is calculated for each location as follows: ${DALY}_{ay,\;py,\;ey}=\sum_{i=1}^{20}{a_{i,y}*p}_{y}*e_{i,y}$, in the expression, ${DALY}_{ay,\;py,\;ey}$ denotes the estimated DALY attributable to the age structure, total population size, and age-specific DALY rate in y. The term $\;a_{i,y}$ represents the proportion of the population in age group i among the 20 predefined age groups in year y; $p_{y}$ denotes the total population in year y, and $e_{i,y}$ indicates the age-specific DALY rate for age group i in year y [29]. The Bayesian age-period-cohort (BAPC for short) model is a statistical method used to analyze and predict the incidence, mortality and other time series data of diseases. This model combines three factors: age, period and cohort, and uses the Bayesian method for modeling and inference. It is widely applied in fields such as epidemiology, demography and health economics. This approach implements an APC model within the Integrated Nested Laplace Approximation (INLA) framework for Bayesian inference. Compared with traditional Markov Chain Monte Carlo (MCMC) methods, INLA substantially reduces computational burden and circumvents issues related to MCMC mixing and convergence, while retaining high estimation accuracy [30]. This study applied a first-order random-walk (RW1) prior based on restricted natural cubic splines to smooth age, period, and cohort effects. Weakly informative priors were specified, with the precision parameter modeled using a Gamma distribution. Bayesian inference was conducted using INLA framework, and model adequacy was assessed using the Deviance Information Criterion (DIC). This study assessed the performance of the BAPC model using time-series data on the disease burden of lung cancer attributable to diet low in fruits in China and the global from 1990 to 2011 as the training set and from 2012 to 2021 as the test set. Model performance was evaluated using the mean absolute percentage error (MAPE). A MAPE value within the ranges of (0, 5%), [5%, 10%), [10%, 20%), [20%, 50%), and [50%, 1] indicated high precision, high accuracy, good performance, reasonable performance, and poor performance, respectively [31]. This study uses R version 4.3.2 (the R foundation for statistical computing) and the BAPC model to predict the disease burden of lung cancer attributable to diet low in fruits in China and the global over the next 25 years.

## 3. Results

### 3.1. The burden of lung cancer attributable to diet low in fruits in 1990 and 2021

Tables 1 and 2 display the burden of lung cancer attributable to diet low in fruits in China and the global in 1990 and 2021. In 2021, the mortality numbers of lung cancer attributable to diet low in fruits in China and the global were 19,147.7 (95%UI: 9,773.4–30,001.5) and 66,045.5 (95%UI: 34,005.9−97,033.5); DALY was 445,041.3 (95%UI: 224,120.4−701,687.5) and 1,611,267.0 (95%UI: 828,053.6−2,347,369.5) person-years. Additionally, the EAPC of age-standardized mortality rate was −3.0 and −1.9; DALY rate was −3.5 and −2.2, respectively. From 1990 to 2021, the mortality number of lung cancer attributable to diet low in fruits among male increased by 1.08 times, while the DALY increased by 0.87 times. Moreover, during the period from 1990 to 2021, the EAPC of age-standardized mortality and DALY rates among male were both below −2, at −2.78 and −3.29, respectively. From 1990 to 2021, the number of mortality and DALY among female increased by 1.07 times and 0.84 times, respectively. The EAPC of age-standardized mortality and DALY rates were −3.50 and −4.00, respectively. The decrease in mortality number, DALY, and age-standardized rates of lung cancer attributable to diet low in fruits was faster among female than among male.

**Table 1:** Analysis and temporal trends of mortality, DALY and age-standardized rates of lung cancer attributable to diet low in fruits in China in 1990/2021.

	1990			2021		1990-2021 EAPC (95%CI)
		Number (95%UI)	ASR. per100000 (95%UI)	Number (95%UI)	ASR. per100000 (95%UI)	
**Mortality**	**Both**	17,695.0 (8,642.0, 27,166.2)	2.2 (1.1, 3.3)	19,147.7 (9,773.4, 30,001.5)	0.9 (0.5, 1.5)	−3.0 (−3.2, −2.9)
	**Male**	12,037.7 (5,521.0, 19,208.2)	3.2 (1.5, 5.1)	13,082.2 (6,441.7, 21,523.4)	1.4 (0.7, 2.3)	−2.8 (−2.9, −2.6)
	**Female**	5,657.4 (2,778.2, 8,883.0)	1.4 (0.7, 2.1)	6,065.4 (2,949.1, 9,857.4)	0.6 (0.3, 0.9)	−3.5 (−3.8, −3.3)
**DALY**	**Both**	514,951.8 (252,181.5, 772,460.5)	56.3 (27.5, 85.0)	445,041.3 (224,120.4, 701,687.5)	21.0 (10.6, 32.9)	−3.5 (−3.7, −3.4)
	**Male**	352,768.6 (167,108.9, 564,172.2)	78.6 (36.8, 125.1)	308,390.9 (150,698.9, 502,491.9)	30.2 (14.9, 49.2)	−3.3 (−3.4, −3.2)
	**Female**	162,183.1 (80,506.0, 256,471.8)	35.4 (17.6, 55.5)	136,650.4 (65,605.9, 222,253.5)	12.6 (6.1, 20.5)	−4.0 (−4.3, −3.8)

**Abbreviations: ASR, age-standardized rate. EAPC, estimated annual percentage change. UI, uncertainty interval. CI, confidence interval.**

**Table 2:** Analysis and temporal trends of mortality, DALY and age-standardized rates of lung cancer attributable to diet low in fruits in the global in 1990/2021.

	1990			2021		1990-2021 EAPC (95%CI)
		Number (95%UI)	ASR. per100000 (95%UI)	Number (95%UI)	ASR. per100000 (95%UI)	
**Mortality**	**Both**	51,621.4 (25,769.8, 75,860.2)	1.3 (0.7, 1.9)	66,045.5 (34,005.9, 97,033.5)	0.8 (0.4, 1.1)	−1.9 (−2.0, −1.8)
	**Male**	37,726.7 (18,866.6, 56,539.4)	2.1 (1.1, 3.1)	44,328.3 (22,872.1, 64,126.7)	1.1 (0.6, 1.6)	−2.2 (−2.2, −2.1)
	**Female**	13,894.7 (7,255.5, 20,690.6)	0.7 (0.3, 1.0)	21,717.2 (11,100.4, 32,326.8)	0.5 (0.2, 0.7)	−1.3 (−1.4, −1.2)
**DALY**	**Both**	1,435,375.1 (721,498.8, 2,119,984.3)	34.4 (17.2, 50.7)	1,611,267.0 (828,053.6, 2,347,369.5)	18.5 (9.5, 26.9)	−2.2 (−2.3, −2.1)
	**Male**	1,059,478.5 (534,061.4, 1,599,531.8)	53.6 (27.0, 80.7)	1,097,919.6 (566,432.7, 1,597,498.8)	26.5 (13.7, 38.5)	−2.5 (−2.5, −2.4)
	**Female**	375,896.6 (196,209.1, 560,465.6)	17.2 (9.0, 25.6)	513,347.4 (261,759.0, 754,495.9)	11.3 (5.7, 16.6)	−1.6 (−1.8, −1.5)

**Abbreviations: ASR, age-standardized rate. EAPC, estimated annual percentage change. UI, uncertainty interval. CI, confidence interval.**

**Fig 1:**
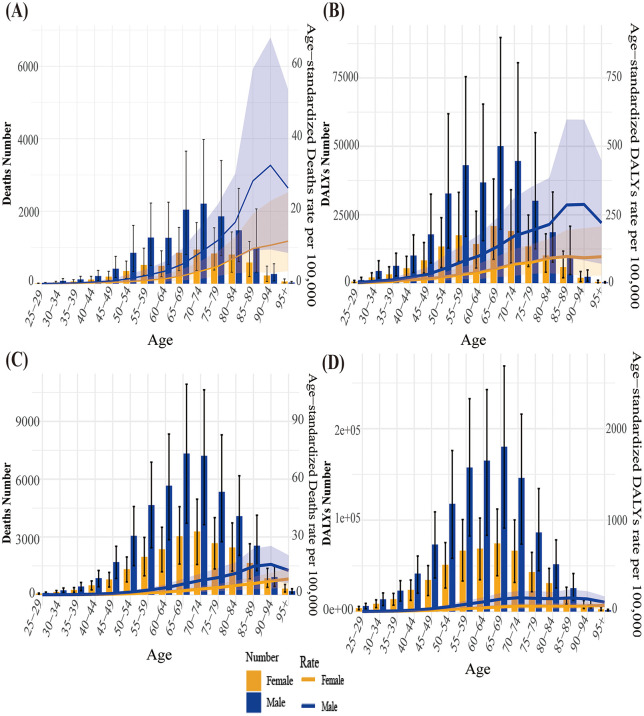
The burden of lung cancer attributable to diet low in fruits by age and gender in China and the global in 2021. (A) China mortality. (B) China DALY. (C) Global mortality. (D) Global DALY. The shaded areas represent the upper and lower limits of the 95% uncertainty interval (95% UI). DALY, disability-adjusted life year.

Fig 1 displays the burden of lung cancer attributable to diet low in fruits by age group in China and the global in 2021. Looking at different age groups, the overall trend of the burden of lung cancer attributable to diet low in fruits in China increases with age. Moreover, within different age groups, the mortality number also increases with age, reaching a peak at 70–74 years. The mortality rate of lung cancer attributable to diet low in fruits in male shows an upward trend first and then a downward trend with age, reaching a peak at 90–94 years and then gradually decreasing. In contrast, the mortality rate of lung cancer attributable to diet low in fruits in female gradually increases with age. Similarly, the age-standardized DALY rate shows a similar trend to the mortality rate in terms of gender and age group. The DALY rate of lung cancer attributable to diet low in fruits increases first and then decreases with age, gradually increasing after the age of 30 and reaching a peak at 65–69 years. The mortality number, mortality rate, DALY number and DALY rate of lung cancer attributable to diet low in fruits are all higher in male than in female.

### 3.2. Trends in the burden of lung cancer attributable to diet low in fruits in China and the global from 1990 to 2021

**Fig 2:**
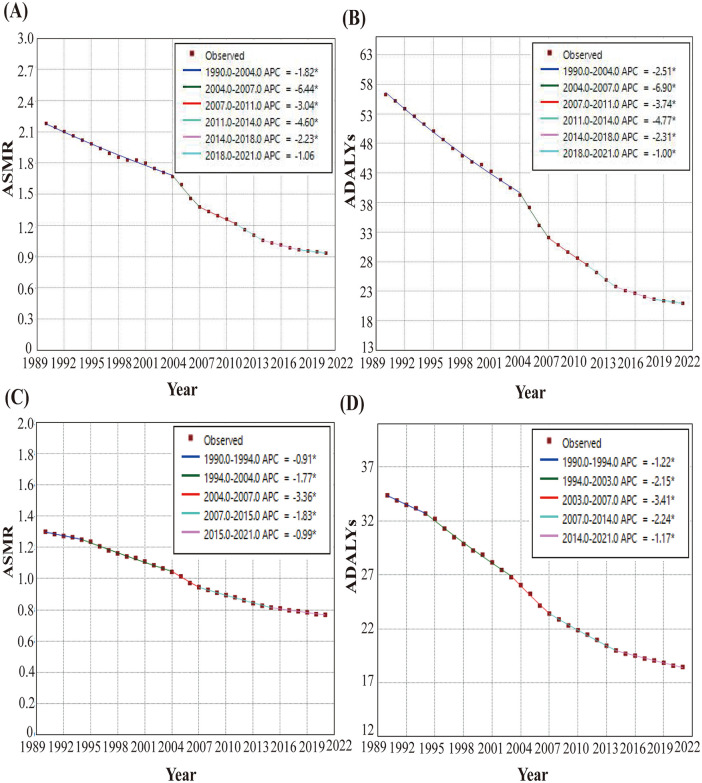
Results of joinpoint regression model for age-standardized rates of the burden of lung cancer attributable to diet low in fruits in China and the global from 1990 to 2021. (A) China ASMR. (B) China ASDR. (C) Global ASMR. (D) Global ASDR. ASMR, age-standardized mortality rate. ASDR, age-standardized disability-adjusted life year rate. * mean *P* < 0.05 and significant results.

The joinpoint regression model results of the burden of lung cancer attributable to diet low in fruits in China and the global, from 1990 to 2021 show that during the 31-year observation period, the trend of mortality of lung cancer attributable to diet low in fruits experienced five turning points. Over the period 1990–2021, both China and global ASMR and ASDR for lung cancer attributable to diet low in fruits showed significant decreases (all *P* < 0.05) (Fig 2).

### 3.3. Decomposition analysis of the burden of lung cancer attributable to diet low in fruits in China and the global from 1990 to 2021

**Fig 3:**
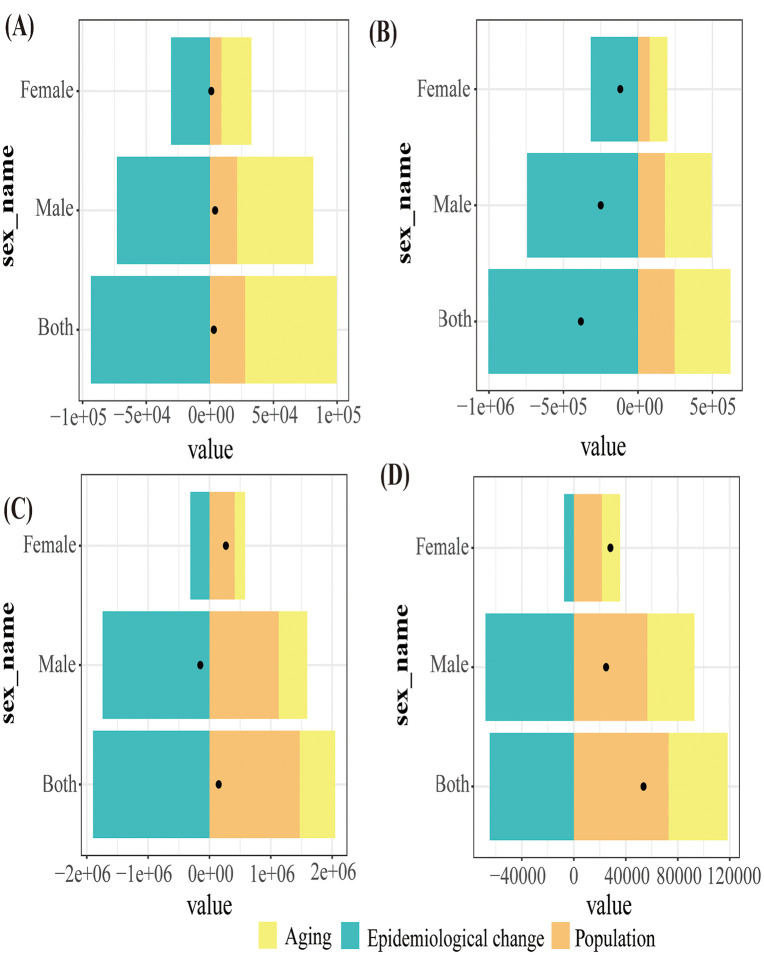
The relative contributions of population aging, population growth and epidemiological changes to the burden of lung cancer attributable to diet low in fruits in China and the global from 1990 to 2021. (A) China mortality. (B) China DALY. (C) Global mortality. (D) Global DALY. The black dot represents the overall value of change contributed by all 3 components. For each component, the magnitude of a positive value indicates a corresponding increase in lung cancer attributable to diet low in fruits to the component; the magnitude of a negative value indicates a corresponding decrease in lung cancer attributable to diet low in fruits to the component. DALY, disability-adjusted life year.

Population aging and growth have collectively contributed to the rise in the absolute mortality number of lung cancer attributable to diet low in fruits, whereas epidemiological transitions have played a crucial role in mitigating this increase. Over the past three decades, these demographic shifts have also been the primary drivers of the rise in DALY associated with diet low in fruits for lung cancer. Nonetheless, favorable epidemiological developments such as improvements in dietary patterns, the implementation of effective tobacco control policies, and advancements in medical technologies have helped curb the growth of DALY (Fig 3). Although the aging population and demographic expansion have intensified the overall burden of lung cancer, these public health and medical improvements have partially offset the negative effects of demographic changes by reducing individual-level risks of disease and mortality.

### 3.4. A predictive analysis of the burden of lung cancer attributable to diet low in fruits in China and the global

**Fig 4:**
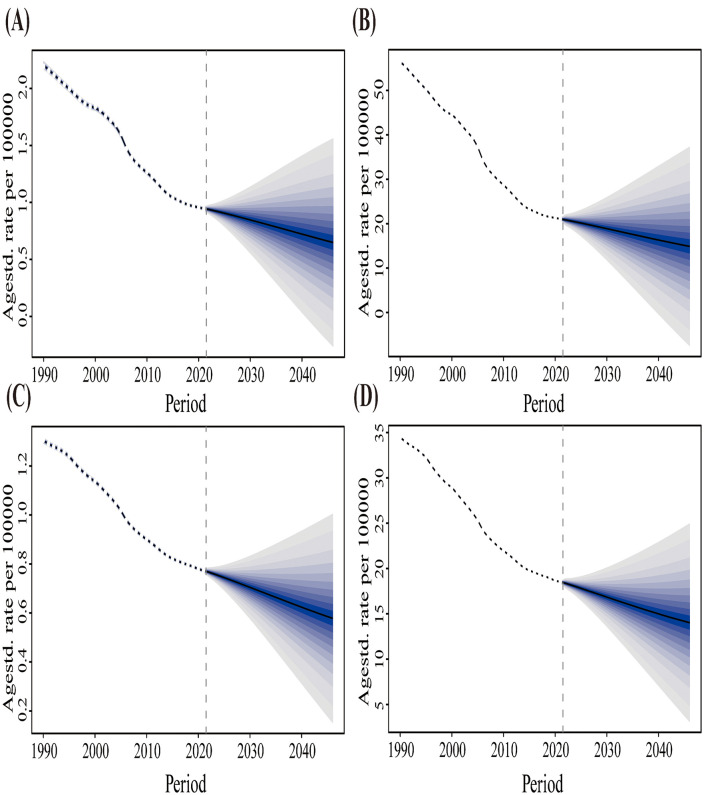
Observed and predicted trends of lung cancer mortality and DALY rates attributable to diet low in fruits in both sexes in China and the global from 1990 to 2046 using the BAPC model. (A) China ASMR. (B) China ASDR. (C) Global ASMR. (D) Global ASDR. The dotted lines represent the actual observed values from the GBD database, and the solid black lines represent the predicted average values. The shaded areas in the figure represent the uncertainty intervals, indicating that the age-standardized rate may fluctuate significantly by ±1% each year. Each shaded area corresponds to a 1% change. ASMR, age-standardized mortality rate. ASDR, age-standardized disability-adjusted life year rate. BAPC, Bayesian age-period-cohort.

Based on the data of mortality and DALY rates of lung cancer attributable to diet low in fruits in China and the global, from 1990 to 2021, the BAPC model was used to predict the mortality and DALY rates of lung cancer attributable to diet low in fruits by gender in China and the global from 2022 to 2046. In China, the prediction results show that the mortality rate is expected to decrease from 0.95 per 100,000 cases in 2021 to 0.65 per 100,000 cases in 2046, a decrease of 31.58%; The DALY rate is expected to decrease from 21.07 per 100,000 cases in 2021 to 14.90 per 100,000 cases in 2046, a decrease of 29.28%; Globally, the prediction results show that the mortality rate is expected to decrease from 0.77 per 100,000 cases in 2021 to 0.58 per 100,000 cases in 2046, a decrease of 24.68%; The DALY rate is expected to decrease from 18.53 per 100,000 cases in 2021 to 14.02 per 100,000 cases in 2046, a decrease of 24.34% (Fig 4). The disease burden of lung cancer attributable to diet low in fruits has shown a consistent annual decrease. Trend prediction based on historical data and statistical methods, rather than causal inference.

## 4. Discussion

This study presents a comprehensive analysis of the most recent data on the temporal trends of lung cancer mortality and DALY attributable to diet low in fruits in China and the global, stratified by age group and year, from 1990 to 2021. The paper findings reveal that during this period, the number of lung cancer mortality and DALY attributable to diet low in fruits increased to varying extents in both sexes. However, the age-standardized rates exhibited a consistent decrease across different time periods and birth cohorts. Luo et al.’s study indicates that male experience a greater burden of diet-related cancers compared to female, while female tend to place more emphasis on health and body shape management [32]. Unhealthy lifestyle choices among male, such as the excessive consumption of processed meats and insufficient intake of fruits and vegetables, may result in a decrease in dietary quality, thereby elevating the risk of lung cancer [17]. A prospective cohort study has demonstrated that adherence to a low-fat diet can reduce the risk of lung cancer in individuals aged 55 and older, particularly among smokers. Conversely, a high intake of saturated fatty acids may elevate the risk of lung cancer, especially small cell lung cancer, in middle-aged and elderly populations [8].Therefore, it is recommended that targeted health intervention strategies be developed for male, as well as for middle-aged and elderly populations, accompanied by proactive health education and awareness campaigns.

The findings of this study indicate that between 1990 and 2021, the mortality number, mortality rate, DALY, and DALY rate associated with diet low in fruits for lung cancer in China and the global all exhibited a downward trend. As fruit consumption in the diet increases, the risk of lung cancer progressively decreases [11,14,33]. Additionally, research by Yin et al. further substantiates that moderate intake of citrus fruits can significantly lower the risk of lung cancer [34]. Simultaneously, a balanced diet abundant in vegetables, fiber, vitamin C, fish, and white meat can also contribute to a reduction in lung cancer risk [12,35]. Notably, the carbohydrates and fiber found in fruits, vegetables, and whole grains offer protective benefits. In contrast, refined carbohydrates present in processed foods (such as soft drinks) [36] and the consumption of beer have been associated with an increased risk of lung cancer [13]. A meta-analysis has demonstrated that adherence to the Mediterranean diet pattern significantly reduces the risk of lung cancer. This dietary pattern is characterized by an abundance of plant-based foods, such as fruits and vegetables, and is rich in dietary fiber and bioactive compounds, including flavonoids, carotenoids, vitamins C, A, and E, folic acid, and coumarin [11]. Meanwhile, a prospective cohort study has shown that adhering to a healthy plant-based diet may reduce the mortality rate of lung cancer [37]. Optimizing dietary patterns and enhancing awareness of healthy eating are crucial in mitigating the burden of lung cancer. It is essential for government and public health institutions to strengthen public education and awareness campaigns on the importance of healthy eating, encouraging individuals to scientifically increase their intake of fruits, vegetables, fish, and white meat to reduce the risk of lung cancer. Concurrently, appropriate food policies should be developed to improve the accessibility and affordability of nutritious foods, thereby fostering the overall health of the population.

The predictive results of this study indicate that the disease burden of lung cancer attributable to diet low in fruits has shown a consistent annual decrease. According to the BAPC model prediction results that by 2046, the age-standardized mortality and DALY rates of lung cancer attributable to diet low in fruits in China and the global will decrease by 31.58%, 24.68%, 29.28%, and 24.34%, respectively. This aligns with the findings of Li L et al. which indicate that the burden of lung cancer attributable to diet low fruits in China and the global has been decreasing annually and is expected to continue decreasing over the next 15 years [8]. The disease burden of lung cancer attributable to diet low in fruits has been decreasing annually, likely due to factors such as economic development, the widespread dissemination of nutrition education, and the implementation of policies promoting healthy eating. Since the introduction of the first nutrition pyramid by the United States Department of Agriculture in 1992, there has been a growing emphasis on balanced diets, which has facilitated a shift toward more balanced and health-conscious dietary patterns [8,38]. A recent report indicates that adopting the flexible, plant-based Planetary Health Diet (PHD), reducing global food loss and waste by 50%, implementing sustainable ecological agricultural practices, and halting the conversion of intact ecosystems into agricultural land could concurrently improve population health and restore planetary health. The report estimates that by 2050, these strategies could ensure an adequate food supply for a projected global population of 9.6 billion. Moreover, transforming global food systems and dietary patterns in this manner could substantially reduce the incidence of diet-related chronic diseases, including cardiovascular disease, diabetes, and cancer, preventing approximately 15 million premature deaths annually [39]. The Mediterranean diet is grounded in the traditional dietary practices of countries bordering the Mediterranean Sea, such as Greece, Italy, and Spain. It is characterized by high consumption of fresh fruits, vegetables, whole grains, legumes, nuts, and olive oil; moderate intake of fish and poultry; and limited consumption of red meat and dairy products. This dietary pattern is not only rich in flavor but is also widely recognized as one of the healthiest diets globally due to its balanced nutritional profile. Moreover, evidence from a meta-analysis indicates that adherence to the Mediterranean diet is significantly inversely associated with the risk of lung cancer [11]. China has progressively enhanced dietary risk management within its cancer prevention and control policies. In alignment with the “Healthy China 2030 Planning Outline”, “Implementation Plan for Cancer Prevention and Control under the Healthy China Initiative (2023-2030)”, “Chinese Dietary Guidelines (2022)”, and “China’s Nutrition Improvement Program”, the country has implemented a range of evidence-based interventions. Furthermore, improvements in socioeconomic conditions have increased the public’s access to high-quality food resources, thereby fostering healthier dietary behaviors and contributing to a reduction in cancer risk. In recent years, China has achieved substantial economic and social advancement, accompanied by continuous enhancement of medical infrastructure. Concurrently, widespread health education initiatives have been implemented at the national level. Collectively, these developments have played an important role in promoting cancer prevention and supporting the adoption of healthier lifestyles [27]. These include limiting the consumption of red and processed meats, increasing the intake of whole grains and dietary fiber, and advocating for low-salt, low-sugar, and low-fat diets. These measures aim to systematically improve the dietary structure of the population. Additionally, efforts have been made to strengthen nutrition and health education to promote healthy eating behaviors, reduce cancer risk, improve the health literacy of the public, and effectively mitigate the strain on medical resources. Nevertheless, policy and social factors may exert potential influence on the observed trends; however, the current models are unable to independently quantify their effects. These potential impacts require validation through further empirical investigation.

Dietary factors may modulate lung cancer development through multiple biological pathways. Fruits and vegetables are essential components of the human diet, providing vitamins, minerals, phytochemicals, and dietary fiber, all of which may contribute to lung cancer prevention. The potential protective effects of fruit and vegetable consumption have been widely discussed, with proposed mechanisms including the regulation of DNA methylation, mitigation and repair of DNA damage, promotion of apoptosis, and induction of phase II detoxification enzymes [40].The protective association is largely attributed to the abundant phytochemicals present in these foods—such as cyanogenic glycosides, indoles, and flavonoids—which can modulate various tumor-related signaling pathways. Through these mechanisms, such compounds may inhibit tumor cell proliferation, induce apoptosis, and ultimately reduce lung cancer risk [41–43].

This study is the first to leverage the latest data from the GBD 2021 to assess the burden of lung cancer attributable to diet low in fruits in China and the global. However, it is not without limitations. Firstly, the GBD database exhibits heterogeneity in terms of data quality and completeness, which may result in inaccurate or incomplete estimates of the disease burden in China, potentially impacting the representativeness of the findings. The uncertainty inherent in GBD data, potential variations arising from model revisions across different versions, and the limited availability of data in low-resource regions may all affect the accuracy of the estimates. These factors could introduce biases or increase uncertainty in certain estimates; therefore, the results should be interpreted with caution. Secondly, dietary factors encompass a range of components, including diet low in fruits and diet low in vegetables, diet low in fiber, diet high in processed meat and diet high in red meat. However, due to the limitations inherent in the GBD database, this study was restricted to evaluating only the impact of diet low in fruits on the burden of lung cancer. Although the GBD database provides comprehensive global data, these estimates may require adjustments when applied to specific countries or regions to ensure that policy recommendations are both targeted and effective. Additionally, this analysis focused solely on the impact of diet low in fruits on mortality and DALY, without considering their influence on lung cancer incidence. Simultaneously, regarding attribution and causal inference, it should be noted that the GBD data are derived from model-based associations; therefore, these represent ecological model-based correlations rather than definitive causal estimates. The BAPC model predictions do not explicitly account for potential future intervention strategies, shifts in the food supply system, or national nutrition policy adjustments. Accordingly, the projected outcomes should be interpreted primarily as indicators of long-term trends and policy foresight rather than as precise estimates of absolute burden levels. Lastly, due to the use of only national-level data, the study did not explore regional differences, including variations between provinces, urban and rural areas in China.

## 5. Conclusions

In conclusion, the burden of lung cancer attributable to diet low in fruits in China and the global has shown signs of alleviation, with expectations that the mortality and DALY rates of lung cancer resulting from diet low in fruits will continue to decrease over the next 25 years. Nevertheless, the overall situation remains substantial and warrants ongoing attention. Accordingly, the paper suggestions that public health policies be strategically directed towards male and the elderly, with an emphasis on promoting health education initiatives to encourage the promotion of adequate intake of fruits, vegetables, fish, and white meat consumption. Future research should focus on further elucidating the causal relationship between diet low in fruits and lung cancer, as well as identifying effective intervention strategies to mitigate the lung cancer burden. Such efforts would provide a solid scientific foundation for the development of precise and targeted public health policies.
